# Anti-thrombotic effect of a factor Xa inhibitor TAK-442 in a rabbit model of arteriovenous shunt thrombosis stimulated with tissue factor

**DOI:** 10.1186/s13104-018-3886-4

**Published:** 2018-10-30

**Authors:** Emiko Shinozawa, Masaki Kawamura

**Affiliations:** Research, Takeda Pharmaceutical Company Limited, 26-1, Muraokahigashi 2-chome, Fujisawa, Kanagawa 251-8555 Japan

**Keywords:** Factor Xa inhibitor, Arteriovenous shunt model, Tissue factor

## Abstract

**Objective:**

Arterial thrombosis is triggered by tissue factor, which is a transmembrane glycoprotein can be released into the blood circulation after plaque rupture. Animal models with reflecting ruptured plaque lesions will be useful to understand efficacy of anticoagulant. In this study, we sought to improve a common arteriovenous shunt model in rabbits, aiming for a model of thrombosis stimulated with tissue factor, and to investigate the anti-thrombotic effect of a direct factor Xa inhibitor TAK-442 in the model.

**Results:**

In the model where thrombus was stimulated with a thrombogenic silk thread soaked with recombinant human tissue factor, thrombus formation was significantly reduced by TAK-442 at more than 37.5 µg/kg, accompanied with prolonged plasma hemostatic parameters. Although efficacious doses of anti-coagulants in ordinary arteriovenous thrombosis models are widely reported to be higher than those in venous thrombosis models, TAK-442 showed its efficacy in the present arteriovenous shunt thrombosis model, with equivalent sensitivity in a previously reported venous model. TAK-442 might be effective under conditions thrombus formed is more influenced by tissue factor pathway.

## Introduction

Anti-thrombotic agents have been tested on their efficacies and effects on bleeding in various animal thrombosis models; however, a trigger and location of thrombus are different for each model and it is difficult to compare preclinical findings between drugs and studies [1]. In addition, some anti-thrombotic agents, including some inhibitors of activated factor Xa (FXa), have been already approved for clinical use, whereas others, including TAK-442, could not show their efficacies in clinical studies [2, 3]. TAK-442 is an oral direct FXa inhibitor. FXa is located on the crossing point of the extrinsic and intrinsic components of the coagulation cascade. TAK-442 inhibits human FXa with an inhibitive constant (Ki) of 1.8 nM and showed the good antithrombotic effect to bleeding risk in both arterial and venous experimental settings [4, 5]. Although, in a clinical trial for acute coronary syndrome, TAK-442 showed a significant increased risk of bleeding with a lack of the efficacy outcomes [2], its possibility, such as one as a combination therapy with antiplatelet agents, has not elucidated yet.

Besides, as the extrinsic coagulation initiator and a primary trigger of arterial thrombosis, we focused on tissue factor, a transmembrane glycoprotein which can be released into the blood circulation after plaque rupture [6]. It was reported that, in almost all experimental arterial and venous thrombosis models, tissue factor have great effect on thrombus formation [7]. For the arteriovenous shunt thrombosis model, we consider that there is a room to improve it by a method of highlighting the extrinsic coagulation initiator tissue factor. In the present study, we aimed for a more physiologically relevant arteriovenous shunt model with thrombus more influenced by tissue factor, and investigated the effects of TAK-442 in the model to partially address its potential efficacy of thrombus inhibition.

## Main text

### Methods

We modified the previously reported arteriovenous shunt model in rabbits [8, 9]. Ten-week-old male Japanese White rabbits were purchased from Kitayama Labes Co., Ltd. (Japan) and individually housed in metal cages in an air-conditioned room, under a 12:12-h light–dark cycle with food and water provided ad libitum. A total of 10 rabbits were used at 11- to 14-week-old (weighing 2.2–3.0 kg) to develop the modified model, and 4 rabbits of them were used to evaluation of TAK-442 in the model. Briefly, in anesthetized rabbits, the carotid artery and the jugular vein in a rabbit were carefully isolated from surrounding tissues and cannulated with a shunt catheter. The shunt (27 cm in length) was constructed with polyethylene catheters (PE 240; inner diameter, 1.67 mm, and SP 95; inner diameter, 1.2 mm; Natsume Seisakusho Co., Ltd., Japan) as follows. The sections (each 3-cm-long SP 95) were inserted into a rabbit carotid artery and jugular vein; then, they were connected to the central part of the shunt (a 15-cm-long PE 240) having intermediate portions at both sides (each 3-cm-long SP 95). The polyethylene tubes were connected with each other using silicone tubes. Thrombus formation was induced by setting a thrombogenic 7-cm-long silk thread soaked with recombinant human tissue factor (Dade Innovin, Siemens, Marburg, Germany) placed inside the central part of the shunt. The selective FXa inhibitor TAK-442, 1-(1-{(2S)-3-[(6-chloro2-naphthyl)sulfonyl]-2-hydroxypropanoyl}piperidin-4-yl)tetrahydropyrimidin-2(1H)-one, which was synthesized at Takeda Pharmaceutical Company Limited. (Osaka, Japan), or vehicle control (5% *N*,*N*-dimethylacetamide/5% polyethylene glycol/90% saline) was administered as a bolus (two-thirds of total dose) followed by a constant infusion (one-third of total dose) via femoral vein. Drug administration was initiated 30 min prior to shunt placement and continued for 30 min during the experimental protocol in an ascending dose order of 18.75, 37.5, and 75 μg/kg. The silk thread was removed 15 min after blood began to circulate through the shunt at each dose, and the thrombus-coated thread was weighed. For 5 min during each thrombus formation, blood was collected from femoral artery in plastic syringes containing 3.8% sodium citrate (1:9 citrate/blood, vol/vol) for measuring plasma hemostatic parameters. At the end of each experiment, the animals were sacrificed by injection of a lethal dose of pentobarbital sodium.

For plasma hemostatic parameters, prothrombin time (PT), activated partial thromboplastin time (APTT) and anti-FXa activity were measured. Platelet-poor plasma was prepared by centrifugation (13,000 rpm) of plasma obtained from individual rabbits for 10 min at 4 °C. PT and APTT were measured with an automatic blood coagulometer (STA compact; Diagnostica Stago, Inc., France) by using clinical assay kits (Roche Diagnostics K.K., Japan). Anti-FXa activity was also measured with a clinical assay kit (Testzym Heparin S) by using 96-well microplates. Briefly, buffer solution (80 μL) and platelet-poor plasma (10 μL) were mixed with 1 U/mL antithrombin III (10μL) at room temperature for 5 min. After the addition of 7.1 mkat/mL of FXa solution (50 μL), the reaction was initiated with the addition of 0.75 mg/mL of chromogenic substrate solution (100 μL). Three minutes later, 50% acetic acid solution (50 μL) was added, and the O.D. at 405 nm was then measured with a microplate reader. Anti-FXa activity (% inhibition) was calculated as follows: % of inhibition = (1 − O.D. of plasma sample/O.D. of normal rabbit plasma) × 100.

Data are expressed as mean ± standard error of the mean. Differences between groups were assessed using a one-tailed Williams’ test. Differences were considered significant at P ≤ 0.025.

### Results

**Fig. 1:**
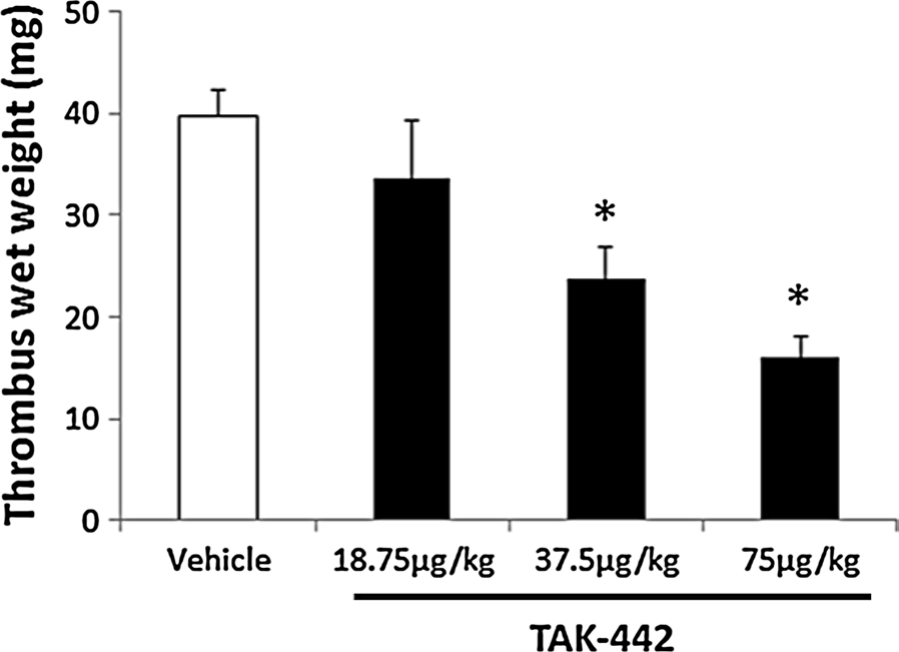
Antithrombotic effect of TAK-442 in a modified rabbit arteriovenous shunt thrombosis model. In a rabbit model of arteriovenous shunt thrombosis stimulated with a thrombogenic silk thread soaked with recombinant human tissue factor, the thrombus-coated thread was weighed 15 min after shunt placement at each dose. The drug was administered as a bolus (two-thirds of total dose) followed by constant intravenous infusion (one-third of total dose) for 30 min prior to shunt placement. Data are expressed as mean ± standard error of the mean; n = 4 animals per treatment group. *P ≤ 0.025 versus vehicle (one-tailed Williams’ test)

In the rabbit model of arteriovenous shunt thrombosis stimulated with tissue factor, we first confirmed that thrombus formation constantly repeated at least four times and the weights of thrombus formed were similar in all times (data not shown). In the model, the thrombus formation was dose-dependently and significantly reduced by TAK-442. Specifically, TAK-442 at total doses of 37.5 and 75 µg/kg reduced thrombus formation by approximately 40% (P ≤ 0.025, one-tailed Williams’ test) and 60% (P ≤ 0.025, one-tailed Williams’ test), respectively (Fig. 1). TAK-442 treatment to the model also showed a dose-dependent increase in plasma anti-FXa activity as well as prolongation of PT and APTT (Table 1).

**Table 1:** Effects of TAK-442 on plasma hemostatic parameters in a modified rabbit arteriovenous shunt thrombosis model

	Control (basal value)	TAK-442 (μg/kg)			
		0 (vehicle only)	18.75	37.5	75
Anti-FXa activity (% inhibition)	13 ± 2.5	12 ± 1.8	17 ± 3.8	26 ± 3.6	45 ± 4.7
PT (% of control)	100 ± 0.62	102 ± 2.2	105 ± 2.0	109 ± 2.5	116 ± 3.7
APTT (% of control)	100 ± 3.3	106 ± 6.5	119 ± 5.4	121 ± 5.9	129 ± 7.5

In a rabbit model of arteriovenous shunt thrombosis stimulated with tissue factor, plasma anti-FXa activity and clotting times were measured. Blood was collected for 5 min during each thrombus formation. The drug was administered as a bolus (two-thirds of total dose) followed by constant intravenous infusion (one-third of total dose) for 30 min prior to shunt placement. Data are expressed as mean ± standard error of the mean; n = 4 animals per treatment group

### Discussion

As widely reported, higher doses of anticoagulants were needed to exert their anti-thrombotic effects in arteriovenous models than in venous models; for instance, which differed at least three to four times in efficacy of some other FXa inhibitors [1, 10]. However, in the present study, TAK-442 showed its significant efficacy even at 37.5 µg/kg, which is equivalent to the effective dose that we previously found in a rabbit venous thrombosis model with endothelial damage and blood stagnation [4]. Further, consistent with a finding in the venous model [4], the 50% inhibition of plasma FXa activity was found at about 75 µg/kg of TAK-442, suggesting a good sensitivity of the present modified arteriovenous model to efficacy of TAK-442. Related to this, another finding was reported by our previous study: the combined therapy with TAK-442 and antiplatelet agents in rats reduced the formation of arterial thrombosis without additional significant increase in bleeding time [11]. Taken together, although further investigations remain to be conducted to obtain clearer insights into the following possibility: FXa inhibition by TAK-442 may exert its anti-thrombotic property at a lower dose under conditions that the extrinsic coagulation pathway is activated relatively more than the platelet and other pathways, and one of which might be a clinical situation while being treated with an antiplatelet agent.

### Conclusions

In the present study, a modified rabbit model of arteriovenous shunt thrombosis was successfully worked by highlighting the extrinsic coagulation initiator tissue factor. This model might be an additional useful tool for preclinical assessment of anticoagulants targeting arterial thrombosis to understand their efficacies. More important is the suggestion that TAK-442 might be effective to suppress thrombus formation under conditions where tissue factor pathway works relatively stronger than the other pathways.

## Limitations

TAK-442 was tested only in the arteriovenous shunt model of thrombosis stimulated with tissue factor; therefore, the data of no setting of the thrombogenic thread soaked with tissue factor with or without TAK-442 should be obtained and examined. It will give more information on the relationship between the stimulation of tissue factor and changes in plasma hemostatic parameters, and on whether and how TAK-442 involves intrinsic and extrinsic pathways.

## Abbreviations

APTT: activated partial thromboplastin time
FXa: activated factor Xa
PT: prothrombin time
